# The psychological and cognitive factors causing college students’ demotivation to learn English in China

**DOI:** 10.3389/fpsyg.2022.890459

**Published:** 2022-08-12

**Authors:** Xiaobin Ren, Jirapa Abhakorn

**Affiliations:** ^1^National Institute of Development Administration, Bangkok, Thailand; ^2^Hubei Business College, Wuhan, China

**Keywords:** demotivation to learn English, grounded theory, structural equation modeling, psychological and cognitive factors, English teaching and learning

## Abstract

Demotivation is one of the important factors causing students’ failure in learning a language. To explore the psychological and cognitive factors causing college students’ demotivation to learn English in China’s universities and to investigate the relations among these internal factors, this study constructed a shopping cart model by applying grounded theory method and tested the model by using structural equation modeling. This study found three paths underlying students’ demotivation to learn English, originating from large discrepancy between students’ actual and required positioning of English learning, low required positioning of English learning and low value of English learning in students’ minds. Based on these findings, this study gave some pedagogical implications for English teaching.

## Introduction

Learning motivation is vitally important for the successful acquisition of English among language learners (Oxford and Shearin, 1994), therefore motivation to learn English has long been a heated research topic in the field of second language acquisition (SLA; Dörnyei, 1990), especially after Gardner and Lambert (1972) proposed integrative and instrumental motivation. But in language teaching practice, numerous students suffer from gradually decreasing investment and engagement in the process of English learning (see Dörnyei and Ushioda, 2011; Pishghadam et al., 2019a). This phenomenon also increasingly attracted language researchers’ attention in recent years because of the problems resulting from it. For example, Falout et al. (2009) proposed English learning demotivation could cause numerous problems among EFL learners and their teachers, including learners’ unfavorable behaviors, negative attitudes, undesired learning results, language teachers’ demotivation and decreased class dynamics. Zeynali et al. (2019) investigated learning motivation of Ph.D., MA, and BA students’ and found demotivation was one of the most strong factors predicting their bad language learning results. Shaikholeslami and Khayyer (2006) and Boonchuayrod and Getkham (2019) also found students’ demotivation was negatively related with their English achievements and learning results.

Moreover, and practically, demotivation to learn English among college students in many EFL countries is not a rare phenomenon (e.g., Trang and Baldauf, 2007; Sakai and Kikuchi, 2009; Kim, 2015; Boonchuayrod, 2019). In China, many college students tend to gradually suffer from demotivation to learn English after they enter universities (Li and Zhou, 2017; Li, 2021). This serious problem poses a challenge for a great number of EFL teachers and learners there. Therefore, many researchers (Sakai and Kikuchi, 2009; Ghadirzadeh et al., 2012; Boonchuayrod, 2019; Wang and Guan, 2020) thought more demotivational studies should be conducted among English learners to solve students’ demotivation and hence improve English teaching and learning efficiency.

Currently, there are some studies conducted to explore students’ demotivation of English learning, and several research found students’ internal factors could be the potential reasons for their demotivation to learn English (e.g., Kaivanpanah and Ghasemi, 2011; Kim, 2015; Akay, 2017). However, there are some problems in the existing studies. For instance, the psychological and cognitive factors causing students’ demotivation of English learning explored in some research were seemingly scattered and independent from each other, and few studies were conducted linking students’ those internal factors. Besides, most of the existing research did not differentiate the internal and external factors. With these considerations, this study focused solely on internal perspective and investigated those psychological and cognitive factors underlying Chinese college students’ demotivation to learn English and constructed a theoretical model to demonstrate the relations among those internal factors.

This research systematically reviewed the studies of demotivation to learn English, broke the research routine of emphasizing external factors in most studies, and exclusively investigated internal factors for students’ demotivation. Those internal factors could enrich the understanding of college students’ demotivation to learn English, and hence help to overcome demotivation. In addition, a theoretical model to explain students’ psychological and cognitive factors for their demotivation to learn English was constructed in this study. The model creatively related different internal factors underlying demotivation, rather than only listing the factors and ignoring the relations among them. It could provide comprehensive theoretical explanations for students’ inner processes underlying their demotivation of learning English.

## Literature review

### Definition of demotivation

Motivation provides language learners with fundamental trigger to learn (Çankaya, 2018). Without sufficient motivation, individuals could hardly achieve their language learning goals even with appropriate curriculum and teaching methods (Boonchuayrod, 2019). As the dark side of motivation, demotivation was also named as passive motivation or negative motivation (Boonchuayrod and Getkham, 2019; Pishghadam et al., 2021). The definition of demotivation was discussed by many researchers in SLA field. Dörnyei and Ushioda (2011, p. 139) once defined demotivation as “specific external forces that reduce or diminish the motivational basis of a behavioral intention or an ongoing action.” Nonetheless, some researchers (e.g., Sakai and Kikuchi, 2009; Clare et al., 2019, p. 66) did not agree with Dörnyei and Ushioda (2011) definition of demotivation, and they included both internal and external factors when they were investigating demotivation. In addition, Sakai and Kikuchi (2009, p. 58) even though the definition of Dörnyei and Ushioda (2011) demotivation was contradictory because they still include internal factors, such as “lack of confidence” and “negative attitude,” as the sources of demotivation in their research. This means that, apart from external factors, internal factors should also be considered when investigating college students’ demotivation to learn English.

### Demotivation in SLA studies

Currently, external factors causing students’ demotivation to learn English were frequently investigated in existing research. Teacher-related factors, teaching contents and materials, class characteristics and environment were the most frequently reported ones. For example, Sakai and Kikuchi (2009) investigated the demotivating factors in Japanese high schools and found teachers’ competence and teaching styles was one of the two most significant demotivators. Wang and Guan (2020) delved into the demotivation factors of learning English in Chinese context and found teacher-related factors were also the most influencing demotivator. Pishghadam et al. (2021) specifically examined the role of teachers’ stroking behaviors and concluded that teachers’ inappropriate stroking behaviors could cause students’ passive motivation for EFL learning. In addition, teaching contents and materials were also found a demotivator in numerous studies. For instance, Kikuchi and Sakai (2009) topped English textbooks in all the external factors influencing Japanese high school students’ demotivation to learn English. Li and Zhou (2017) found teaching material was also the top demotivator among all external factors. On top of that, numerous studies also investigated the influences of class characteristics and environment on students’ demotivation. Çankaya (2018) investigated the demotivation factors in vocational schools and found class characteristics and environment had more negative influences than teacher factors and teaching materials on students’ motivation to learn a foreign language. Besides, educational levels or grades were also considered as a significant correlating factor for students’ demotivation of English learning in Korean elementary school (Kim, 2011; Kim and Seo, 2012).

In addition to those external factors, some studies also found students’ internal reasons could be the causes for their demotivation of English learning. For instance, Trang and Baldauf (2007) found apart from teacher-related factors, students’ past English learning experience, attitudes to English learning, and self-esteem might also be the underlying reasons for their demotivation. Sakai and Kikuchi (2009) agreed that students’ past failure in English learning could be a potential demotivator hindering their progress. Ghadirzadeh et al. (2012) found lack of perceived individual competence and intrinsic motivation were among the factors causing Iranian students’ demotivation. Besides external factors, Akay (2017) also found lack of interests in English and negative attitudes toward English teachers were the internal demotivating factors.

### Research problems and aims

Although some studies investigated students’ internal reasons for their demotivation to learn English, those different factors were scattered and independent from each other in most of the existing studies. Numerous studies only listed the potential internal factors causing demotivation, but few researchers considered the relations between or among different psychological and cognitive factors or linked them after those various internal factors were discovered. For example, Wang and Littlewood (2021) explored the factors underlying EFL learners’ demotivation in Hong Kong master students, and listed several categories causing students’ demotivation, including “failure experience, lack of confidence, lack of interests in English,” etc., but they did not notice the potential relations between or among those underlying factors. This might be problematic given people’s psychology and cognition are usually correlated with each other (see DiLorenzo et al., 2007; del Bosque and Martín, 2008; Su and Shum, 2019). In addition, because of the influence of the definition of demotivation proposed by Dörnyei and Ushioda (2011), some studies (e.g., Kikuchi and Sakai, 2009) only focused on external factors when they were investigating demotivation to learn a foreign language, but studies focusing on students’ internal factors were rarely conducted. In this study, the psychological and cognitive factors causing college students’ demotivation were focused and explored, and the relations among those factors and students’ demotivation were also investigated. To accomplish the research aims, this study proposed 2 research questions to guide the whole study:

What are the cognitive and psychological factors causing students’ demotivation to learn English and how do these factors cause demotivation?

## Materials and methods

### Instruments

In this study, semi-structured interviews and questionnaires were used to collect data.

To ensure the validity of semi-structured interviews, two experts in English teaching (both with a doctoral degree) were invited to evaluate the interview questions designed by the researchers, and the interview questions were revised accordingly. In the end, an interview guideline (see Appendix 1) consisting of eight questions were formulated. This guideline was then applied into one-on-one trial interviews among three college students, and it demonstrated the interview guideline was effective and could generate desired results.

In addition, questionnaires were also applied in this research. The questionnaire used in this research was a seven-point Likert Scale with 30 items, which was utilized to test the constructs in the theoretical model constructed in this research. When the questionnaire was being designed, two experts in questionnaire designing provided their suggestions for questionnaire revision. A pilot study was conducted among 86 college students in China’s universities, and the item analysis, reliability analysis and validity analysis demonstrated the questionnaire could be used as an instrument in this study.

### Student sampling and data collection

#### Student sampling and data collection for constructing model

Theoretical sampling method (Strauss and Corbin, 1998; Corbin and Strauss, 2014) was applied to select college students in China’s universities in this study. The sampling method required researchers to collect and analyze data initially and then, based on the needs of data enrichment, determine where to collect data and what data to collect next. Theoretical sampling is a recurrent process and should not end before the data is saturated (Glaser and Strauss, 1967, p. 45; Corbin and Strauss, 2014, p. 150). Based on the guideline of theoretical sampling, the researchers in this study one-on-one and face-to-face interviewed 23 college students (for details, see Table 1) in several universities in the capital of a central province of China according to the questions in the interview guideline (Appendix 1) until the data was saturated.

**Table 1:** Interviewees’ basic information.

Student code	Gender	Grade (year)	Major
SYR	Male	Second	Hospitality management
MJS	Female	First	Film production
LSS	Male	Third	Accounting
HJX	Female	Fourth	Finance
YYL	Male	Third	Management
WMQ	Male	First	Tourism management
TWJ	Male	Second	Photography
ZT	Male	Second	Electronic information
CLM	Female	Third	Management
TXY	Female	Third	Film production
JNY	Male	Third	Digital media
YXJ	Female	Second	Financial management
ZZY	Male	First	Visual communication design
WWQ	Male	First	Computer science
HJS	Male	First	Data science
THS	Female	Third	Management
ZSL	Male	Second	Photography
WJW	Female	Fourth	Hospitality management
LCR	Male	Third	Hospitality management
JBN	Female	Second	Tourism management
DML	Male	Fourth	Computer science
HZJ	Male	Second	Data science
CYL	Female	Third	International economics and trade

The interviews were conducted in students’ canteens, classrooms, and coffee shops near the campus. An informed consent form was given to each interviewee before the interview started. All interviews were recoded, and those recoded interviews were transcribed into Chinese texts through https://www.iflyrec.com/. After that, those transcribed Chinese texts were proofread by the researchers.

#### Student sampling and data collection for testing model

To test the theoretical model constructed through grounded theory, this study adopted random sampling method and chose 10 college classes (for details, see Table 2) from different universities.

**Table 2:** Information of students filling in questionnaire.

Class code	Student count	Demotivated student count	Major	Grade	University code
1	45	33	Tourism management	Second	PRU1
2	36	26	Management	Third	OPU1
3	32	25	Financial management	Second	GPU1
4	39	32	Accounting	Second	GPU1
5	35	23	Hospitality management	Fourth	PRU1
6	42	33	Photography	Second	GPU2
7	31	29	Film production	Third	OPU2
8	41	26	Computer science	Second	OPU1
9	46	31	Digital media	Fourth	PRU2
10	33	28	Accounting	Second	PRU1
Total	380	286	---	---	---

In China, public universities are ranked higher than private ones in university rankings, and generally those public universities listed in the “double first-class” university project (Ministry-of-Education, 2017) boasted better student sources and education quality than those excluded from the project. Based on these considerations, this study classified China’s universities into three categories: private university (PRU), ordinary public university (OPU), and good public university (GPU) and randomly selected classes in the above three categories of universities.

Students in the above-mentioned classes were asked to fill in questionnaires within 20 min in their English classes guided by their English teachers. Totally, 380 students successfully submitted their questionnaires, and 286 students thought they suffered from demotivation of English learning. The information of these classes and demotivated students is displayed in Table 2.

### Data analysis

#### Analysis of interview data

This study analyzed the interview data with grounded theory method (Corbin and Strauss, 2014). According to Corbin and Strauss (2014), grounded theory method has three stages, including open coding, axial coding, and selective coding.

##### Open coding

Open coding coded the texts based on lines or sentences. Sentences and lines could be coded into various concepts. Then, similar concepts were integrated, and categories emerged.

##### Axial coding

Axial coding compared and integrated different categories developed in open coding and explored the relations between those categories and then developed main categories.

##### Selective coding

Selective coding further integrated the main categories developed in axial coding and selected a core category from them.

The three data coding stages were not a one-time process, but rather a recurrent one, in which the data were coded, compared, integrated, and categorized recurrently. In this study, data coding software NVivo was applied in the data analysis process to improve the data coding efficiency.

#### Analysis of questionnaire data

With the data from 286 questionnaires of the demotivated students, this study tested the grounded theory model by using the method of structural equation modeling (SEM). SPSS 25 and AMOS 23 were applied as the data analyzing tools.

### Transparency statement

This study used the same interview guideline as that applied in the study of Ren and Abhakorn (2022). In addition, both this study and the previous one adopted same method to analyze interview data (for more details, see, Ren and Abhakorn (2022), pp. 291–295). Nonetheless, the population in the present study was different from that in their study. This study only focused on non-English major college students, while their study did not differentiate English and non-English majors.

## Results

### Grounded theory model development

The next three sub-sections display some examples and elaborate the results of open coding, axial coding, and selective coding.

#### Open coding

Open coding in this study was to code the interview data line by line without preconceptions. In the open coding stage, interviewees’ answers were coded into concepts, and those concepts were further coded into categories.

Firstly, 23 transcribed files were imported into NVivo and then was analyzed by open coding method. In the open coding stage, the interview data were abstracted three times. Firstly, 1,456 nodes were generated among the 23 files in NVivo, and 65 concepts emerged. Those concepts were then further coded into 19 categories. Table 3 showcases the conceptualization and categorization of some examples in the interview data.

**Table 3:** Open coding process.

Transcribed text (translated)	Conceptualization	Categorization
Long English sentences are too difficult for me because the structure is very complex.	Difficulty in analyzing sentence structure	Poor grammar
For example, in my English textbook, I do not know more than half of the words’ meaning.	Unfamiliar with many words	Poor vocabulary
English teachers speak English too fast. I cannot follow them.	Difficulty in following teachers	Poor listening ability
I have never learned phonetic symbol before. My English teacher cannot understand me.	Shortage of speaking knowledge	Poor speaking ability
New horizon college English textbook is too difficult for us students majoring in arts.	Difficult textbooks	Difficult teaching materials
College English Test-4 (CET-4) is too difficult for me.	Difficult English tests	Difficult tests
Our English teachers always gives us detailed review guidelines before the final test.	Providing hints for test	Decreased test requirements
For me, some grammar rules and vocabulary have been learnt in middle school. We can learn them by ourselves.	Repetitive grammar rules and vocabulary	Easy teaching contents
I cannot remember English words. I have tried many times, but I always forgot in the end.	Cannot remember words	Low vocabulary learning efficiency
I have learned English for many years without many achievements. I do not think I can make much progress anymore.	Little progress in opinion	Low expectancy of English learning
You know, in our school, there is no opportunities speaking English.	Little use in campus	Limited use chances
As for photography major, professional courses are more important than English.	Less important than another course	Limited significance
I had a sense of failure of English learning.	Sense of failure of English learning	Sense of failure
I am afraid of having English classes, because …	Afraid of English classes	Afraid
I felt nervous when I have to speak English.	Nervous for speaking English	Nervous
I hate English classes and teachers…. I hate English textbooks.	Hate elements of English learning	Negative emotions
My enthusiasm for learning English is not as strong as when I was a freshman.	Weaker enthusiasm for learning English	Decreased enthusiasm
My time spent on learning English dropped sharply compared with when I just entered university.	Time on learning English dropped	Dropped learning time
I have almost given up learning English, because…	Give up learning English	Give up learning

#### Axial coding

Axial coding in this study aimed to integrate related categories developed in open coding by exploring the relations among them, and then develop main categories (Strauss and Corbin, 1998). In this coding stage, six main categories were developed by integrating related categories in open coding. Table 4 demonstrates the axial coding results.

**Table 4:** Axial coding results.

Main categories	Further categorization	Categories	Connotation of main categories
Low actual positioning	Weak language foundation	Poor grammar	Students’ English abilities was weak in their minds.
		Poor vocabulary	
		Poor listening ability	
		Poor speaking ability	
Required positioning	High requirements	Difficult teaching materials	Students’ perceptions about outside English learning requirements.
		Difficult tests	
	Low requirements	Decreased test requirements	
		Easy teaching contents	
Low self-efficacy	Undesired results	Low vocabulary learning efficiency	Students’ expectancy about the possibility of achieving their goals.
	Low expectancy	Low expectancy of English learning	
Low value	Limited value	Limited use chances	Low value means the worth or significance of learning English in students’ minds is low.
		Limited significance	
Negative affects	Negative affects	Sense of failure	Negative affects refer to students’ bad feelings, emotions, moods, attitudes, etc.
		Afraid	
		Nervous	
		Negative emotions	
Demotivation	Negative learning behaviors	Decreased enthusiasm	Demotivation means gradually decreasing investment in the process of English learning among college students in this study
		Dropped learning time	
		Give up learning	

#### Selective coding

In this study, selective coding had 2 aims: further integrated the main categories developed in axial coding and selected a core category from them. After the analysis of the main categories and the transcribed data, large actual-required positioning discrepancy emerged. Besides, a core category emerged after the cognitive maps (Zhang, 2011) of the 23 participants were drawn and compared. According to Chaney (2010), cognitive maps could be utilized to display the causes and effects in the interview data and to demonstrate interviewees’ mental processes clearly and logically. To draw the cognitive maps of the 23 interviewees, the causes and effects in their remarks were carefully investigated. After that, 23 cognitive maps of concepts were drawn by utilizing the results of concepts (concepts in open coding could be seen in Table 3) in open coding. Then 23 cognitive maps of categories were drawn based on the results of categories (categories in open coding could be seen in Table 3) in open coding by integrating the concepts. In the next step, the 23 cognitive maps of categories were further integrated into one. The integrated cognitive map of categories was further abstracted based on the results of axial coding and selective coding. Eventually, a theoretical model demonstrating students’ psychology and cognition was constructed.

**Figure 1:**
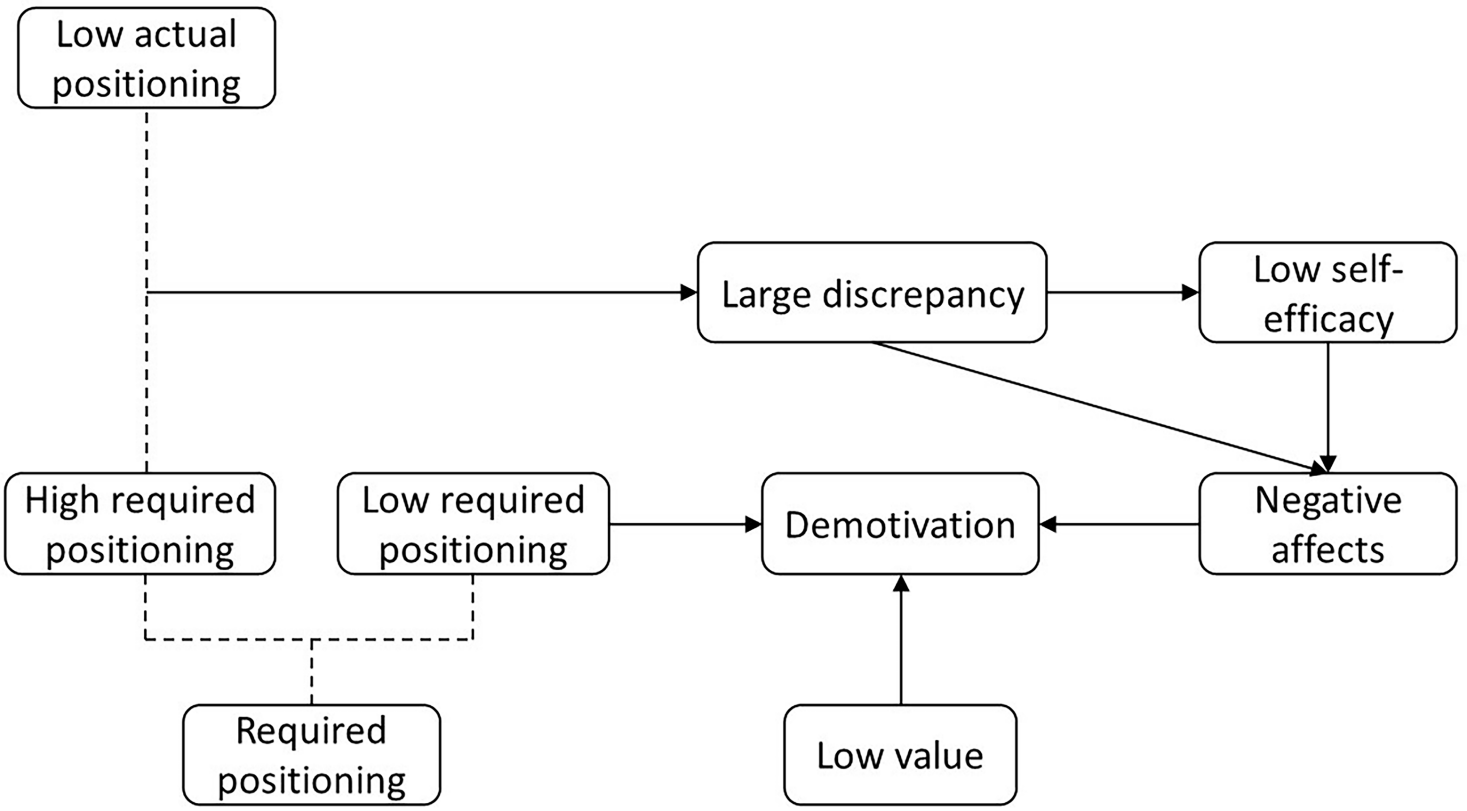
Grounded theory model.

Through the three data analyzing stages of grounded theory method, including open coding, axial coding, and selective coding, this study constructed a shopping cart model (Figure 1). This model displays three paths underlying students’ demotivation to learn English, originating from large discrepancy between students’ actual and required positioning of English learning, low required positioning of English learning and low value of English learning in students’ cognition. The latter two factors, i.e., low required positioning and low value of English learning in students’ minds could directly generate students’ demotivation of English learning, while large discrepancies between actual and required positioning could firstly cause students’ low efficacy, and then negative affects. Alternatively, those discrepancies might directly generate negative affects among college students. Those different negative affects could end up with demotivation of English learning.

### Model testing

This study constructed a shopping cart model to demonstrate the psychological and cognitive factors causing college students’ demotivation to learn English and display the relations among these internal factors and demotivation through analyzing the 23 interviews with students. To test the fitness of the model among large sample size, questionnaires from 286 students were applied.

#### Reliability test

To test the internal consistency of the questionnaire, reliability test was conducted, and the values of Cronbach’s Alpha for each construct are listed in Table 5.

**Table 5:** Values of Cronbach’s α of constructs.

Constructs	Large discrepancy (LD)	Low self-efficacy (LS)	Negative affects (NA)	Low required positioning (LRP)	Low valence (LV)	Demotivation (De)
Cronbach’s α	0.846	0.805	0.790	0.874	0.876	0.908

All the Cronbach’s Alphas were above 0.60, indicating that the questionnaire had reasonable internal consistency.

#### Exploratory factor analysis

SPSS 25 was used to run exploratory factor analysis (EFA) in this study, the values of KMO and Bartlett could be found in following Table 6.

**Table 6:** KMO and Bartlett’s test.

KMO		0.887
Bartlett’s test	Chi-Square	4777.788
	df	435
	Sig.	0.000

The KMO value of the whole questionnaire reached 0.887 and the Sig. ratio was 0.000 < 0.05, which indicated that it was suitable to run principal component analysis. In this study, six components were extracted by choosing correlation matrix and varimax and setting eigenvalue great than 1, and the 6 components could explain 65.17% of the total variance. This indicated that the 6 components could reasonably represent the original data.

#### Confirmatory factor analysis

The structural model should be evaluated after the evaluation of the measurement model (Yang et al., 2016, p. 234). Therefore, convergent validity test was conducted firstly among the six measurement models, and results are displayed in Table 7. All the CRs of the six measure models were above 0.7, and almost all the AVEs were above 0.5, with low self-efficacy near 0.5. These indicators demonstrated that those measurement models had reasonable convergent validity.

**Table 7:** Convergent validity test results.

		Unstd.	S.E.	*t*-value	*p*-value	Std.	SMC	CR	AVE
LD	LD1	1.000				0.682	0.465	0.847	0.527
	LD2	1.040	0.096	10.781	***	0.752	0.566		
	LD3	1.022	0.092	11.067	***	0.779	0.607		
	LD4	0.973	0.092	10.575	***	0.733	0.537		
	LD5	0.927	0.094	9.902	***	0.678	0.460		
LS	LS1	1.000				0.681	0.464	0.805	0.454
	LS2	0.900	0.104	8.681	***	0.617	0.381		
	LS3	1.016	0.110	9.273	***	0.671	0.450		
	LS4	0.987	0.108	9.145	***	0.659	0.434		
	LS5	1.168	0.118	9.884	***	0.735	0.540		
NA	NA1	1.000				0.695	0.483	0.805	0.510
	NA3	1.235	0.118	10.446	***	0.790	0.624		
	NA4	1.055	0.111	9.513	***	0.675	0.456		
	NA5	1.036	0.107	9.666	***	0.689	0.475		
LRP	LRP1	1.000				0.872	0.760	0.838	0.573
	LRP5	0.951	0.059	16.215	***	0.884	0.781		
	LRP3	0.683	0.058	11.701	***	0.647	0.419		
	LRP2	0.658	0.065	10.098	***	0.575	0.331		
LV	LV1	1.000				0.849	0.721	0.881	0.713
	LV5	1.097	0.064	17.015	***	0.931	0.867		
	LV2	0.907	0.063	14.315	***	0.742	0.551		
De	De2	1.000				0.894	0.799	0.892	0.676
	De3	0.849	0.059	14.347	***	0.722	0.521		
	De4	0.987	0.052	19.046	***	0.867	0.752		
	De5	0.891	0.053	16.677	***	0.795	0.632		

*** indicates *p* < 0.001.

Discriminate validity test demonstrated that the square root of every construct’s AVE was higher than the Pearson correlations between the specific construct and others (see Table 8), indicating the measurement models had reasonable discriminate validity.

**Table 8:** Discriminate validity test results.

	AVE	De	NA	LS	LV	LD	LRP
De	0.676	**0.822**					
NA	0.510	0.523	**0.714**				
LS	0.454	0.503	0.575	**0.674**			
LV	0.713	0.504	0.345	0.451	**0.844**		
LD	0.527	0.405	0.540	0.528	0.368	**0.726**	
LRP	0.573	0.402	0.362	0.386	0.243	0.269	**0.757**

Square roots of AVEs are in bold on diagonal, while off diagonal are Pearson correlations of constructs.

#### Model fit

Table 9 demonstrates the major model fit indexes and their corresponding recommended values of good model fit. It demonstrates the model fit index values fall into or were very near the recommend values, indicating that the grounded theory model (shopping cart model) was acceptable.

**Table 9:** SEM indexes and values.

Indexes	Values	Acceptable values
χ^2^	473.454	---
χ^2^/df	1.645	<3.0 (Kline, 2015)
GFI	0.893	>0.80 (Doll et al., 1994)
AGFI	0.870	>0.80 (Doll et al., 1994; Arpaci and Baloğlu, 2016)
CFI	0.949	>0.90 (Kline, 2015)
RMSEA	0.098	<0.10 (Kenny et al., 2015)

#### Hypotheses testing

**Figure 2:**
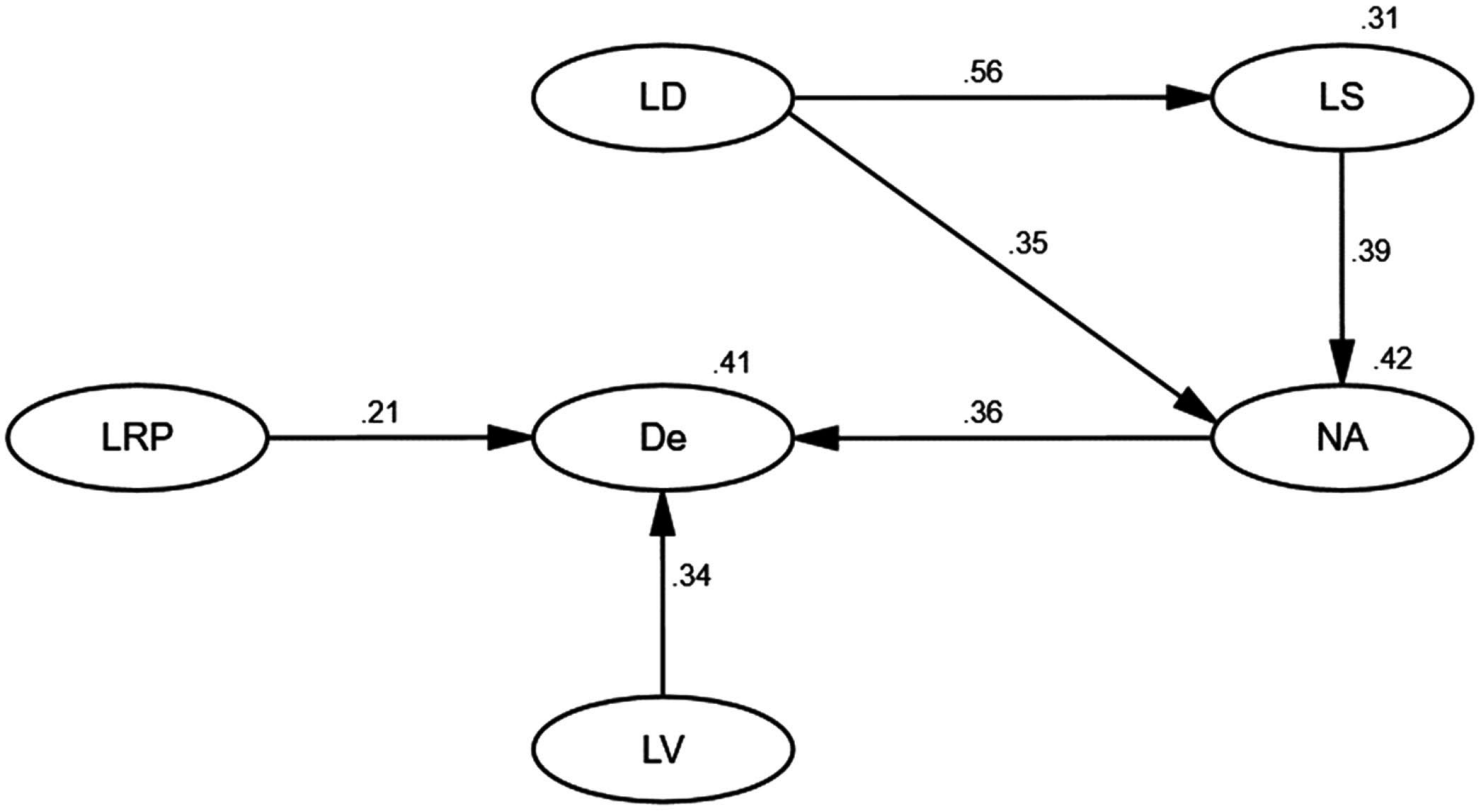
Path coefficients of the structural model (LD refers to large discrepancy between students’ actual and required positioning of English learning; LS refers to low self-efficacy; NA refers to negative affects; De refers to demotivation to learn English; LRP refers to low required positioning; LV refers to low value of learning English.)

Table 10 demonstrates the six hypotheses in the theoretical model, and each path coefficient could be found in Figure 2. In Table 10, the results of tested hypotheses were listed, and it showed that every path in the shopping cart model was significant and acceptable.

**Table 10:** Results of tested hypotheses.

Hypotheses	Simplified relations	Unstd.	Std.	Results
H1: Large discrepancy could reduce students’ self-efficacy.	LD → LS	0.682^***^	0.557	accept
H2: Students’ low self-efficacy could cause students’ negative affects.	LS → NA	0.347^***^	0.393	accept
H3: Large discrepancy could cause students’ negative affects.	LD → NA	0.374^***^	0.345	accept
H4: Negative affects could cause students’ demotivation.	NA → De	0.450^***^	0.362	accept
H5: Low required positioning could cause students’ demotivation.	LRP → De	0.162^***^	0.210	accept
H6: Low valence could cause students’ demotivation.	LV → De	0.307^***^	0.344	accept

*N* = 286.

*** indicates *p* < 0.001.

## Discussion

To explore the psychological and cognitive factors causing college students’ demotivation to learn English in China’s universities, this study constructed a shopping cart model by applying grounded theory method and tested the model by using structural equation modeling. This study found three paths underlying students’ demotivation to learn English, originating from large discrepancy between students’ actual and required positioning of English learning, low required positioning of English learning and low value of English learning in students’ minds. Ren and Abhakorn (2022) conducted a similar study to investigate the internal factors underlying demotivation to learn English. However, there existed differences in the findings of the two studies. One of the most significant differences between the present study and the previous one was in the path originating from large discrepancy to demotivation. The previous study found some students might experience motivation to learn English when they sensed far high English learning requirements, while in the present study, few students mentioned the motivation process before they became demotivation (for more details, see, Ren and Abhakorn (2022), p. 295). This difference might result from the difference of population sampling in two studies. The previous study did not differentiate majors in universities and included several English major participants in that study, while this study only focused on non-English majors. Because English is a vitally important tool for English majors in their future career, they might be firstly motivated and try their best to learn, while for some non-English majors, they might not try to learn but directly display low self-efficacy or negative affects and eventually demotivation. Therefore, considering the differences of learning behaviors, requirements, foundations, goals, etc., among English and non-English majors (Sun et al., 2022), it could be better to differentiate the two groups of students when investigating college students’ demotivation to learn English. Methodologically, although Ren and Abhakorn (2022) constructed a model, it was not tested among large college student sample, thus might not be appropriately be used to explain demotivation among large sample size. However, this study, after developing the shopping cart model, further tested the theoretical model with structural equation modeling among students in different types of universities (i.e., GPUs, OPUs, and PRUs). The mixed method (grounded theory plus SEM) and sample diversity could make the model more acceptable.

This study found numerous students in China’s universities stated that the listening and speaking contents in their English classes were very challenging for them, indicating their required positioning of English listening and speaking in their English classes were very high. Given that grammar-translation teaching method is also popular among second and tertiary English education (Du, 2021), students’ relatively low actual positioning of English listening and speaking may be the results of the popularity of grammar-translation teaching in China’s middle schools and universities. The high required positioning and low actual positioning of listening and speaking made the large discrepancy between them, which was one of the origins of students’ demotivation.

This study found many universities in this study adopted same textbooks, indicating English learning requirements for their students were similar. Hence, their students’ required positioning of English learning shared similarity. However, students in different types of universities turned different attitudes toward their similar required positioning of grammar, vocabulary, and reading skills. In GPUs, students frequently mentioned that the teaching contents, including vocabulary, reading and grammar teaching, were relatively easy for them, which was one of the cognitive origins for their demotivation. However, in PRUs, students usually complained about the difficult teaching contents of grammar and vocabulary. This partly explained the relatively small factor loading of the path from low required positioning to demotivation. In addition, this finding provided psychological and cognitive supports to “i + 1” hypothesis of Krashen (1991). According to this hypothesis, the requirements of language teaching input should be within a certain range. Based on “i + 1” hypothesis, Cheng (2011) further stated that neither “i + 2” (far difficult inputs for students) nor “i + 0” (inputs and students are at the same level) could achieve the best teaching results, but rather the above two kinds of inputs could disturb students’ English learning. In PRUs, many inputs fall into the “i + 2” area, while in GPUs, numerous inputs of grammar, vocabulary and reading knowledge fall into “i + 0” area. The two different inputs could be the potential origins for students’ demotivation to learn English, because too much “i + 2” knowledge is linked with large actual-required positioning discrepancies, while “i + 0” knowledge is related with students’ low required positioning.

Miri and Pishghadam (2021) thought the role of senses should be emphasized when discussing emotioncy because senses connect people with the outside world. Besides, people’s senses were related with their emotional levels, and these senses and emotions could influence people’s motivation levels (Pishghadam et al., 2013; Miri and Pishghadam, 2021). These statements echoed one underlying path in this study: the path deriving from large discrepancy to negative affects, and eventually to students’ demotivation. Specifically, when students sensed the large discrepancies between their actual positioning and the high required position, they might generate negative affects, which could cause demotivation to learn English. Because of the important role of sense and emotioncy in language learning behaviors and in the process of language teaching and learning (for more details, see, Pishghadam et al., 2016, 2019b; Miri and Pishghadam, 2021), hence more research in the future should be conducted to explore the underlying relations among language learners’ sense, emotion, and their demotivated behaviors of learning English.

In addition, this study also found low value of English learning in students’ mind was another origin for students’ demotivation to learn English. This finding afforded cognitive evidence for the expectancy theory of Vroom (1964). According to this theory, people’s evaluation of the value of a certain conduct could affect their motivation level of engaging in the behavior. Students in this study frequently mentioned that their future jobs did not need too much English competence, or English was useless in their daily life. Because of those low value cognitions in their minds, they tended to decrease their motivation of English learning.

## Conclusions and implications

A shopping cart model was constructed to demonstrate the psychological and cognitive factors causing students demotivation and the relations among them. From the model, it can be found that there are three paths underlying CSD, i.e., from large discrepancy between students’ actual and required positioning of English learning, low required positioning of English learning and low value of English learning in students’ cognition. The three paths causing students’ demotivation provided English teachers some implications.

In China’s universities, including GPUs, OPUs and PRUs, the majority of college students’ listening and speaking competences are relatively weak and needed to be improved. Therefore, grammar-translation teaching method should be used combined with other teaching methods to improve students’ English competences comprehensively rather than solely focusing on grammar, vocabulary, and reading skills, etc., while ignoring their English communicative skills.

The teaching of vocabulary, grammar, and reading skills in different types of universities should be differentiated. In some GPUs, the requirements of vocabulary and reading for students might need to be lifted, or attentions paid to those aspects could be shifted to other English skills like listening and speaking. This is because college students in those high-ranking universities (i.e., GPUs) have laid a solid foundation of vocabulary, grammar, and reading by being instructed with grammar-translation teaching method in senior high. But those students’ communicative abilities are still generally weak. Lifting requirements of vocabulary and reading for students could overcome the low required positioning of English learning. While, shifting emphases to their listening and speaking skills could also be one of the choices for overcoming students’ low required positioning of English learning. But in some PRUs, students’ English foundations are weak. Requirements of vocabulary and reading skills could be lowed to avoid too large actual-required positioning discrepancies among students. In addition, decreasing requirements among those weak foundation students might also reduce their pressure and hence avoid demotivation.

More opportunities to use English for college students should be provided in their daily life. Universities could establish connections with international enterprises and provide more intern positions for students working in English-speaking context. Universities or colleges could also employ more teachers and enroll more students from international communities to increase chances of using English in Chinese students’ campus life. Those measures could increase English-speaking opportunities, and thus help to enhance the value of English in students’ cognition.

## Data availability statement

The raw data supporting the conclusions of this article will be made available by the authors, without undue reservation.

## Ethics statement

Ethical review and approval was not required for the study on human participants in accordance with the local legislation and institutional requirements. The patients/participants provided their written informed consent to participate in this study.

## Author contributions

XR designed and wrote the whole article. JA contributed to conception, manuscript revision, proof reading, and approved the submitted version.

## Funding

This research was funded by Hubei Business College with the program name of “A Study of Demotivation to Learn English among College Students in China’s Private Universities” (item code: KY202141).

## Conflict of interest

The authors declare that the research was conducted in the absence of any commercial or financial relationships that could be construed as a potential conflict of interest.

## Publisher’s note

All claims expressed in this article are solely those of the authors and do not necessarily represent those of their affiliated organizations, or those of the publisher, the editors and the reviewers. Any product that may be evaluated in this article, or claim that may be made by its manufacturer, is not guaranteed or endorsed by the publisher.

## Appendix

Appendix 1

**Guideline questions for the semi-structured interview (translated into English).**

1. After you entered university, have you ever experienced demotivation to learn English?

2. When you began your study in the university, how was your motivation of English learning and how is it now?

3. Could you tell me, in detail, about your recent experience of English learning?

4. Could you describe the whole process of your motivation declining of English learning after you entered university?

5. What do you think were the factors leading to your demotivation to learn English?

6. Could you explain how those factors could lead to your demotivation of English learning?

7. Do you have other feelings, emotions, or ideas during your demotivated period of English learning that you want to make up?

8. Do you have some advice for college students to overcome demotivation of English learning?

Appendix 2

**Questionnaire items (translated into English).**

1. The homework assigned by our English teacher was very difficult.

2. The English textbooks were too difficult.

3. English listening was too difficult.

4. English speaking was difficult to be improved.

5. English teachers had too many requirements for us.

6. It is almost impossible for me to have good speaking competence.

7. It is almost impossible for me to have good listening competence.

8. It is almost impossible for me to pass CET-4/6.

9. It is almost impossible for me to have all-English classes.

10. It is almost impossible for me to understand English movies without subtitles.

11. I felt nervous and upset in English class.

12. I felt stressful about English tests.

13. I felt unconfident when I talked with foreigners in English.

14. I was afraid of reading materials written in English.

15. I was worried about making mistakes when speaking English.

16. English is useless in daily life.

17. I have no plan to study overseas.

18. My future work will require no English proficiency.

19. I have no plan to work in international companies.

20. Professional classes are more important than English ones.

21. English teachers would give me high class performance marks even if I did not listen to them carefully.

22. We do not need to attend speaking test in CET-4/6.

23. English teachers will give us hints for the final exam.

24. Many grammatical points taught in college English classes have already been learnt in senior high.

25. English teachers seldom test us at ordinary time.

26. I feel my passion for English learning decreased gradually.

27. I seldom listen carefully in English class.

28. I seldom take the initiative to learn English.

29. I only learn English before the English tests.

30. If possible, I would choose to skip the English classes.

Thanks again for your time and participation!
